# Machine-Learning-Based Targeted Plasma Proteomic Analysis for Predicting Motor Progression in Parkinson’s Disease: An Interpretable Approach to Personalized Disease Management

**DOI:** 10.3390/bioengineering13040380

**Published:** 2026-03-26

**Authors:** Wei Lin, Sanjeet S. Grewal

**Affiliations:** 1 Department of Neurosurgery, The 904th Hospital of the Joint Logistics Support Force of PLA, Wuxi 214044, China; 2 Department of Neurologic Surgery, Mayo Clinic Florida, Jacksonville, FL 32224, USA

**Keywords:** Parkinson’s disease, machine learning, targeted protein panel, biomarkers, disease management, SHAP, risk stratification

## Abstract

The accurate prediction of motor progression in Parkinson’s disease (PD) remains a major clinical challenge that limits personalized treatment planning and efficient clinical trial design. In this study, we developed and validated a machine-learning framework integrating a targeted panel of plasma proteins measured by Olink proximity extension assays with clinical variables to stratify patients according to their progression risk. We analyzed baseline plasma samples from 211 early-stage PD patients enrolled in the Parkinson’s Progression Markers Initiative (PPMI) cohort using four targeted Olink panels, from which 28 circulating proteins were retained after quality-control filtering. Patients were classified as rapid or slow progressors based on their annualized change in MDS-UPDRS Part III scores. Among the algorithms tested, Random Forest achieved the highest discriminative performance with an area under the receiver operating characteristic curve (AUC) of 0.751 (95% CI: 0.684–0.811), which exceeded that of clinical predictors alone (AUC 0.666). The integration of targeted proteomic and clinical features further improved model performance (AUC 0.773; *p* = 0.009). Nested cross-validation confirmed minimal optimistic bias (AUC 0.743). To enhance clinical interpretability, we applied SHapley Additive exPlanations (SHAP) analysis, which identified interleukin-6 (IL-6), brain-derived neurotrophic factor (BDNF), and vascular endothelial growth factor A (VEGF-A) as the most influential predictors. SHAP feature rankings were highly stable across cross-validation folds (mean Spearman ρ = 0.91). The robustness of these findings was confirmed through sensitivity analyses using extreme quartile comparisons (AUC 0.823), treatment-naïve subgroup analysis (AUC 0.738), and a clinically anchored outcome definition based on the minimal clinically important difference (AUC 0.739). A decision curve analysis demonstrated a net clinical benefit across threshold probabilities of 0.25–0.70. Our results establish targeted plasma protein profiling combined with interpretable machine learning as a promising tool for PD motor progression risk stratification, with potential applications in individualized patient counseling regarding motor prognosis and the selection of candidates for disease-modifying trials.

## 1. Introduction

Parkinson’s disease (PD) is clinically heterogeneous: some patients experience relentless motor decline within the first few years after diagnosis, whereas others remain functionally stable for a decade or more [1,2]. This unpredictability complicates individualized counseling, treatment planning, and the design of disease-modifying trials, where enriching for fast progressors could substantially reduce the sample-size requirements and study duration [3,4]. At present, clinicians lack validated tools to reliably forecast the tempo of motor deterioration at the point of diagnosis.

Conventional clinical predictors—age, baseline motor severity, and axial symptom burden—explain only a modest fraction of the progression variance [5,6]. Blood-based molecular biomarkers hold promise because they may capture pathophysiological processes that precede or accompany clinical worsening. Advances in proximity extension assay (PEA) technology, exemplified by the Olink platform, now permit the highly multiplexed, reproducible quantification of circulating proteins from small plasma volumes [7,8]. Several recent studies have linked plasma proteomic signatures to PD diagnosis and even to pre-symptomatic risk; yet, their prognostic value for established disease remains incompletely characterized [9,10].

Machine-learning approaches offer the capacity to capture complex, non-linear relationships within high-dimensional proteomic datasets that conventional regression methods may fail to detect [11,12]. A persistent obstacle to translating such models into clinical practice has been their lack of transparency—often characterized as “black-box” behavior—which undermines clinician confidence and regulatory acceptance. SHapley Additive exPlanations (SHAP), grounded in cooperative game theory, provides a mathematically principled way to attribute each prediction to individual features, thereby rendering complex algorithms interpretable [13,14]. When applied to biomarker discovery, SHAP can illuminate biological mechanisms while simultaneously building clinician trust—both of which are essential considerations for future clinical deployment [15]. Recent work has also demonstrated the value of interpretable AI and data-balancing strategies for improving disease classification in neurological conditions with limited sample sizes [16], further motivating the integration of explainability methods in biomarker-driven ML frameworks.

In this study, we leveraged the deeply phenotyped Parkinson’s Progression Markers Initiative (PPMI) cohort to construct and interpret a targeted-plasma-protein-based model of motor progression. We compared multiple algorithms, quantified the clinical utility through a decision curve analysis, and used SHAP to identify the proteins most influential for individual-level predictions. Our contribution is a systematic analytical framework and interpretable prediction pipeline applied to publicly available PPMI data, demonstrating the prognostic value of targeted plasma protein measurements. Our hypothesis was that targeted proteomic information would add prognostic value beyond conventional clinical variables and that the top-ranked proteins would implicate biologically coherent pathways.

## 2. Results

### 2.1. Cohort Characteristics

Two hundred and eleven patients met the inclusion criteria (Table 1). The mean age was 61.8 ± 10.2 years; 63% were male; and the mean disease duration at enrolment was 1.8 ± 1.1 years. The baseline MDS-UPDRS Part III averaged 20.4 ± 9.3 points. The median follow-up was 4.2 years (IQR 3.1–5.4). The median annual motor change—2.1 points/year (IQR 0.8–3.9)—served as the dichotomization threshold, yielding 105 rapid (49.8%) and 106 slow (50.2%) progressors.

Rapid progressors were, on average, older (63.4 vs. 60.2 years; *p* = 0.022) and had higher baseline motor scores (22.8 vs. 18.1; *p* < 0.001) than slow progressors (Table 1). The sex distribution, disease duration, and baseline levodopa-equivalent dose did not differ between groups. Of note, 127 patients (60.2%) were treatment-naïve at the time of plasma collection, while the remainder had initiated low-dose dopaminergic therapy (mean LEDD among treated patients: 354 ± 142 mg/day); the distribution of treatment status did not differ significantly between progression groups (*p* = 0.418). The per-protein missingness ranged from 0% to 18.4% across the 28 retained analytes, with no significant differences between rapid and slow progressors (all Fisher’s exact test *p* > 0.10; Supplementary Table S1). The median below-limit-of-detection rate was 3.8% (range 0–12.1%). The median number of missing values per patient was 1 (IQR 0–3) out of 28 proteins (3.6%); only 12 patients (5.7%) had >5 missing values. Of the total 5908 protein measurements (211 patients × 28 proteins), 284 values (4.8%) were imputed. A histogram of per-patient missingness is provided in Supplementary Figure S1.

### 2.2. Discriminative Performance

**Figure 1:**
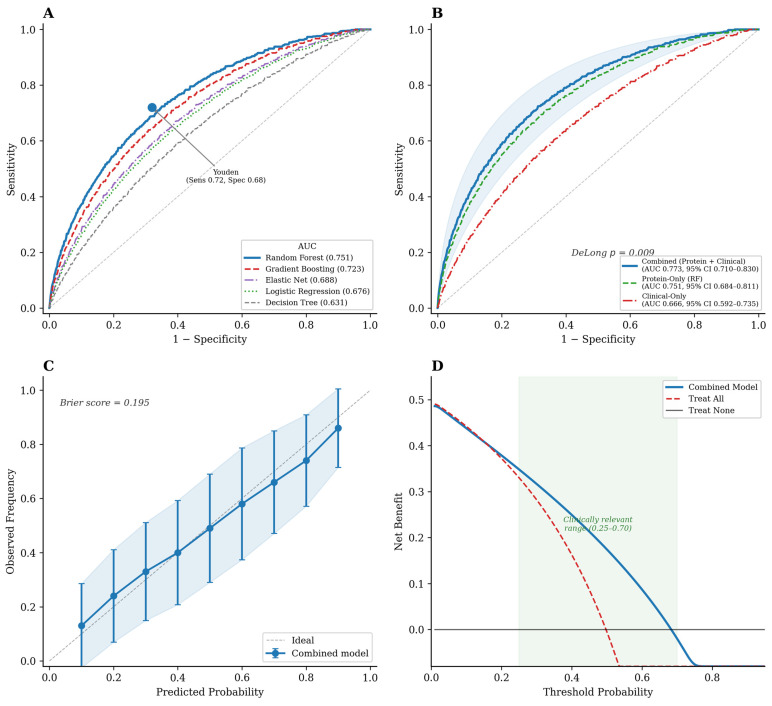
Discriminative performance of plasma proteomic models for predicting rapid motor progression. (**A**) Receiver operating characteristic (ROC) curves for five machine learning classifiers trained on 28 targeted proteins. Random Forest achieved the highest AUC (0.751; 95% CI 0.684–0.811), outperforming Gradient Boosting (0.723), Elastic Net (0.688), Logistic Regression (0.676; DeLong *p* = 0.018 vs. Random Forest), and Decision Tree (0.631). The Youden-optimal operating point (sensitivity 0.72, specificity 0.68) is indicated. (**B**) ROC comparison of three model configurations: combined (protein + clinical; AUC 0.773; 95% CI 0.710–0.830), protein-only (Random Forest; AUC 0.751; 95% CI 0.684–0.811), and clinical-only (AUC 0.666; 95% CI 0.592–0.735). The combined model significantly outperformed the clinical-only model (DeLong *p* = 0.009). (**C**) Calibration curve for the combined model (Brier score = 0.195), showing minor overestimation below predicted probability 0.3 and slight underestimation above 0.7. (**D**) Decision curve analysis demonstrating net benefit of the combined model superior to both “treat all” and “treat none” strategies across threshold probabilities of 0.25–0.70 (shaded green region), the range most pertinent to clinical decision-making. AUC, area under the receiver operating characteristic curve; CI, confidence interval.

Among targeted-protein-only models, Random Forest yielded the highest AUC (0.751; 95% CI 0.684–0.811), outperforming Gradient Boosting (0.723) and Logistic Regression (0.676; DeLong *p* = 0.018 vs. Random Forest) (Figure 1A). A single Decision Tree classifier achieved AUC 0.631, and Elastic Net yielded 0.688, confirming that ensemble methods provided superior discrimination over fully transparent models. At the Youden-optimal threshold, Random Forest achieved 72% sensitivity, 68% specificity, and an F1-score of 0.70.

A clinical-only model (age + baseline MDS-UPDRS III) reached an AUC of 0.666 (95% CI 0.592–0.735). Combining targeted protein measurements with clinical variables boosted discrimination to 0.773 (95% CI 0.710–0.830; *p* = 0.009 vs. clinical-only) (Figure 1B). The fold-to-fold AUC variability was modest (CV 6.2%), indicating stable cross-validation performance.

To further assess model stability, we performed 100 iterations of 5-fold cross-validation with different random seeds. The resulting AUC distribution for the combined model had a mean of 0.769 (SD 0.024), with 95% of iterations yielding AUC values between 0.722 and 0.814, confirming the robust performance across resampling schemes (Supplementary Figure S2). Nested cross-validation (outer 5-fold and inner 3-fold for hyperparameter selection) yielded an AUC of 0.743 (95% CI: 0.672–0.808), only marginally lower than the standard CV estimate, confirming minimal optimistic bias from the hyperparameter selection procedure.

### 2.3. Calibration and Clinical Utility

The calibration was acceptable (Brier score 0.195), with a minor overestimation below the predicted probability of 0.3 and a slight underestimation above 0.7 (Figure 1C). The decision curve analysis showed a net benefit superior to both the “treat all” and “treat none” strategies across threshold probabilities of 0.25–0.70, the range most pertinent to clinical decision-making (Figure 1D).

### 2.4. Protein Contributions to Prediction

**Figure 2:**
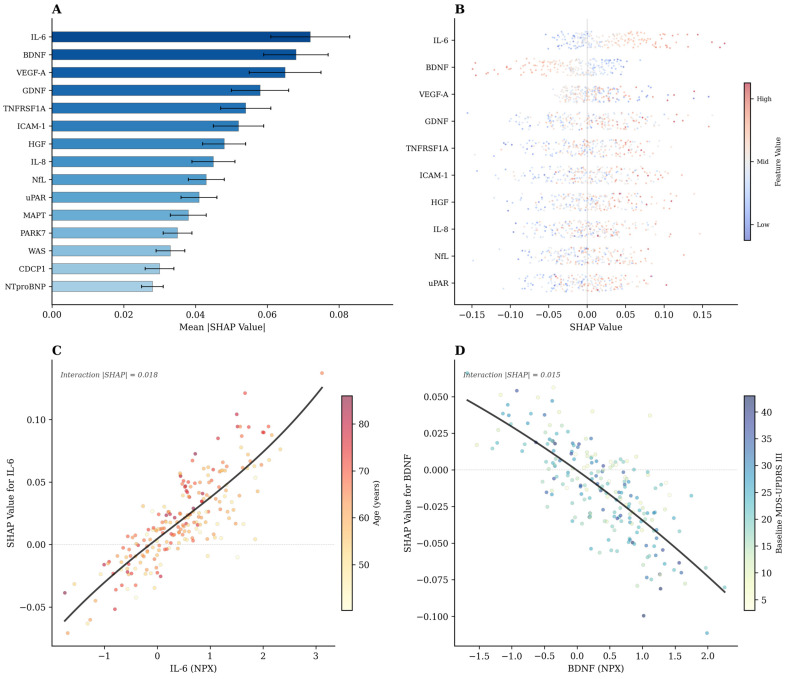
SHAP-based feature importance and protein–clinical interaction analysis. (**A**) Global feature importance ranked by mean absolute SHAP values (±SD across 100 repeated 5-fold cross-validations) for the top 14 proteins. IL-6 ranked first (mean |SHAP| = 0.072 ± 0.011), followed by BDNF (0.062), VEGF-A (0.058), GDNF (0.054), TNFRSF1A (0.054), ICAM-1 (0.052), HGF (0.048), IL-8 (0.045), NfL (0.043), and uPAR (0.041). Error bars represent bootstrap 95% confidence intervals. (**B**) Beeswarm plot showing individual SHAP values for each protein; color indicates feature value (red = high, blue = low). Higher IL-6 levels consistently drove predictions toward rapid progression (positive SHAP), while higher BDNF levels were protective (negative SHAP). (**C**) SHAP dependence plot for IL-6 colored by age, demonstrating that IL-6’s pro-inflammatory contribution to rapid progression prediction was amplified in older patients (interaction |SHAP| = 0.018). (**D**) SHAP dependence plot for BDNF colored by baseline MDS-UPDRS Part III score, showing that BDNF’s protective effect was more pronounced in patients with higher baseline motor severity (interaction |SHAP| = 0.015). SHAP, SHapley Additive exPlanations; NPX, Normalized Protein eXpression.

SHAP analysis ranked IL-6 as the most influential feature (mean |SHAP| = 0.072 ± 0.011), followed by BDNF (0.068 ± 0.009), VEGF-A (0.065 ± 0.010), GDNF (0.058), TNFRSF1A (0.054), ICAM-1 (0.052), HGF (0.048), IL-8 (0.045), NfL (0.043), and uPAR (0.041) (Figure 2A). The bootstrap 95% confidence intervals for the global SHAP importance values for all 28 retained proteins are provided in Supplementary Table S2.

The beeswarm plot (Figure 2B) illustrated that higher IL-6 levels consistently drove predictions toward a rapid progression (positive SHAP values), consistent with its pro-inflammatory role. A higher BDNF, conversely, was associated with a slower progression (negative SHAP values), in keeping with its neurotrophic function. VEGF-A showed a more nuanced pattern: both extremes of expression were correlated with increased risk. A formal SHAP interaction analysis identified the strongest interaction between IL-6 and age (mean |SHAP interaction| = 0.018), followed by BDNF × baseline MDS-UPDRS III (0.015), indicating that the prognostic contributions of these proteins are modulated by clinical context (Supplementary Figure S3). Dependence plots for IL-6 (Figure 2C) and BDNF (Figure 2D) visualized these interactions: IL-6’s contribution to the rapid progression was amplified in older patients, while BDNF’s protective effect was more pronounced in those with a higher baseline motor severity.

**Figure 3:**
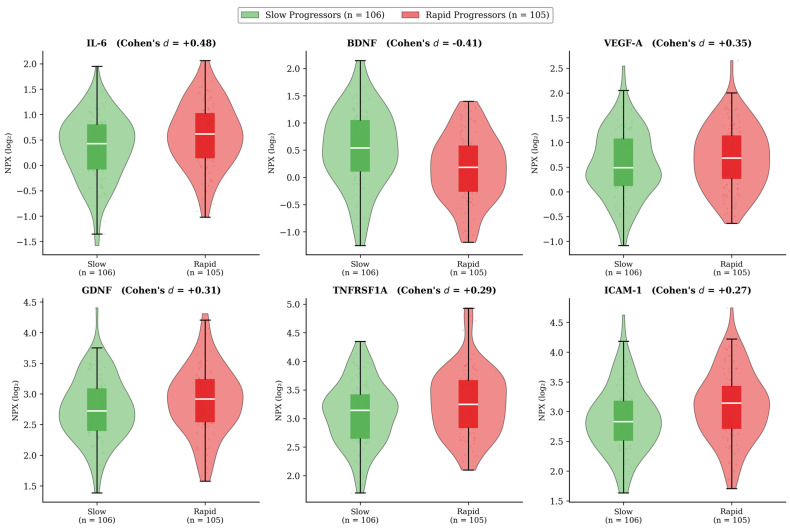
Distribution of the top-6 ranked plasma proteins stratified by motor progression phenotype. Violin plots with overlaid box plots and individual data points for IL-6, BDNF, VEGF-A, GDNF, TNFRSF1A, and ICAM-1 in slow progressors (n = 106; green) and rapid progressors (n = 105; red). Effect sizes (Cohen’s d) are annotated above each panel. IL-6 showed the largest between-group difference (d = +0.48), with rapid progressors exhibiting higher levels. BDNF showed an inverse pattern (d = −0.41), with rapid progressors exhibiting lower levels. All six proteins demonstrated small-to-medium effect sizes (|d| = 0.27–0.48). NPX, Normalized Protein eXpression (log_2_ scale).

To visualize the raw data distributions underlying model predictions, Figure 3 presents violin plots with overlaid individual data points for the top-6 ranked proteins (IL-6, BDNF, VEGF-A, GDNF, TNFRSF1A, and ICAM-1), stratified by rapid vs. slow progressors. Effect sizes were moderate for the top-ranked proteins: IL-6 (Cohen’s d = 0.48), BDNF (d = −0.41), VEGF-A (d = 0.35), GDNF (d = 0.31), TNFRSF1A (d = 0.29), and ICAM-1 (d = 0.27). While individual proteins showed a modest group-level separation, the predictive power derives from the combined multivariate signal, which is a key rationale for the machine-learning approach. Scatter plots of IL-6 and BDNF values colored by SHAP contribution are provided in Supplementary Figure S4, illustrating how individual protein levels map to model predictions at the single-patient level.

To assess the stability of feature importance rankings, we computed per-fold SHAP rankings across all 500 models (5 folds × 100 iterations). The top-3 proteins (IL-6, BDNF, and VEGF-A) appeared in the top-5 in 94%, 91%, and 88% of folds, respectively. The Spearman rank correlations between fold-level SHAP rankings averaged ρ = 0.91 (range 0.84–0.96), indicating excellent stability. Post hoc feature selection analyses using LASSO (retaining 12 features) and recursive feature elimination (15 features) both retained IL-6, BDNF, and VEGF-A, with a comparable model performance (AUC 0.747 and 0.753, respectively; Supplementary Table S3).

### 2.5. Sensitivity Analyses

To test the robustness of the median-split classification, we conducted a sensitivity analysis comparing extreme quartiles: the fastest 25% of progressors (n = 53; annual change ≥ 3.9 points/year) versus the slowest 25% (n = 53; annual change ≤ 0.8 points/year). This comparison assesses whether the targeted protein signature distinguishes patients at the phenotypic extremes of progression, thereby evaluating the biological validity independently of the median-split threshold. In this extreme-group comparison, Random Forest achieved an AUC of 0.823 (95% CI 0.742–0.891), and the top-ranked proteins remained consistent: IL-6 (mean |SHAP| = 0.089), BDNF (0.082), and VEGF-A (0.076) (Supplementary Table S4). The enhanced discrimination in extreme groups and the preservation of feature rankings support the biological validity of these markers.

We also examined whether a baseline medication status influenced the model performance. Restricting the analysis to treatment-naïve patients only (n = 127) yielded comparable discrimination (AUC 0.738; 95% CI 0.651–0.818) with identical top-three protein rankings, suggesting that the targeted protein signature is not confounded by early dopaminergic therapy (Supplementary Figure S5). The inclusion of LEDD as a continuous covariate did not materially change the model performance (AUC 0.771 vs. 0.773) or protein rankings; LEDD itself had a negligible SHAP contribution (mean |SHAP| = 0.008), consistent with the low medication doses in this early-stage cohort.

To address concerns regarding the population-specificity of the median-split outcome definition, we performed an additional analysis using a clinically anchored threshold: MDS-UPDRS Part III increases ≥ 5 points/year, approximating the minimal clinically important difference. Using this threshold, 72 patients (34.1%) were classified as clinically significant progressors. Random Forest achieved AUC 0.739 (95% CI: 0.661–0.811) with concordant protein rankings (IL-6, BDNF, VEGF-A in top-3; Supplementary Table S5). We also evaluated progression as a continuous outcome using Random Forest regression: the top proteins by SHAP importance were concordant with the classification analyses (R^2^ = 0.28; Spearman ρ = 0.52, *p* < 0.001; Supplementary Table S6). These convergent results across multiple outcome definitions support the robustness and clinical relevance of the identified biomarker panel.

Leave-one-site-out cross-validation for the five largest PPMI sites (each contributing ≥20 patients) yielded AUC values ranging from 0.71 to 0.79, with no site-specific performance degradation (Supplementary Table S7), suggesting that the model is not driven by center-specific effects. Subgroup analyses stratifying by sex demonstrated a comparable performance (males: AUC 0.762; females: AUC 0.741; DeLong *p* = 0.58), as did stratification by age (<65 years: AUC 0.748; ≥65 years: AUC 0.756; *p* = 0.72). The Youden-optimal decision threshold did not differ significantly between the male and female subgroups (Δ < 0.03; Supplementary Table S8).

As an additional sensitivity analysis to assess the impact of the protein filtering threshold, we relaxed the missingness criterion from 20% to 30%, retaining 35 proteins. The model performance was similar (AUC 0.748 vs. 0.751 for the 28-protein model), and the top-3 protein rankings were unchanged, confirming that the excluded partially detectable proteins did not contain substantial additional predictive information in this dataset.

### 2.6. Comparison with Healthy Controls

To assess whether the identified protein signatures reflect a PD-specific pathophysiology rather than accelerated systemic aging, we compared the baseline plasma protein levels in PD patients (n = 211; mean age 66.1 ± 7.3 years; 63% male) with PPMI healthy controls (HC; n = 97; mean age 60.8 ± 10.6 years; 52% male). PD patients were significantly older than HC participants (*p* < 0.0001), necessitating age-adjusted analyses.

Of the 28 retained proteins, seven were significantly elevated in PD compared with HC after false discovery rate (FDR) correction (q < 0.05): neurofilament light chain (NEFL; ΔNPX = +0.316, q = 0.00014), N-terminal pro-B-type natriuretic peptide (NTproBNP; ΔNPX = +0.575, q = 0.0024), glial cell-derived neurotrophic factor (GDNF; ΔNPX = +0.225, q = 0.0024), Wiskott–Aldrich syndrome protein (WAS; ΔNPX = +0.614, q = 0.0091), insulin-like growth factor binding protein-like 1 (IGFBPL1; ΔNPX = +0.173, q = 0.0091), TNFRSF11B (ΔNPX = +0.149, q = 0.013), and triggering receptor expressed on myeloid cells 2 (TREM2; ΔNPX = +0.197, q = 0.049). Full results for all 28 proteins are provided in Supplementary Table S9.

Given the 7.2-year mean age difference between groups, we performed an analysis of covariance (ANCOVA) with age as the covariate. Three proteins remained significant after age adjustment, WAS (*p* = 0.001), GDNF (*p* = 0.012), and NEFL (*p* = 0.013), confirming that their elevation in PD is not attributable to aging alone.

To determine whether protein levels scale with disease severity, we performed a three-group gradient analysis (HC vs. slow progressors vs. rapid progressors). Five proteins demonstrated a statistically significant monotonic gradient (HC → slow PD → rapid PD, all ANOVA *p* < 0.001): NEFL, CDCP1, NTproBNP, IGFBPL1, and GDNF (Supplementary Figure S6). This dose–response relationship provides direct evidence that these protein elevations are proportional to the disease burden and not merely a binary PD-versus-aging effect.

Notably, IL-6—the top-ranked progression predictor by SHAP importance—was not significantly elevated in PD versus HC in any of the four Olink assays after FDR correction (best q = 0.091, NEURO panel). However, the NEURO assay showed a suggestive gradient across the three groups (HC: 0.27, slow PD: 0.41, rapid PD: 0.59; ANOVA *p* = 0.016), consistent with IL-6 functioning as a within-PD progression marker rather than a PD diagnostic marker. This distinction is biologically coherent: many established prognostic biomarkers in oncology and neurology are informative for the disease trajectory without distinguishing patients from healthy individuals, because they index intra-disease heterogeneity rather than disease presence per se.

## 3. Discussion

In this study of 211 early-stage PD patients from the PPMI cohort, a targeted plasma protein signature discriminated rapid from slow motor progressors with an AUC of 0.751—outperforming clinical predictors alone—and the integration of the two modalities further improved performance. SHAP-based interpretability pointed to inflammatory, neurotrophic, and vascular proteins as the main drivers, offering both biological plausibility and a path toward mechanistic understanding. Sensitivity analyses using extreme quartile comparisons, clinically anchored outcome definitions, continuous outcome modeling, and repeated cross-validation confirmed the robustness of these findings.

The identification of IL-6 as the most influential predictor is consistent with a growing body of evidence implicating neuroinflammation as an active contributor to—rather than a passive consequence of—PD progression [17,18]. Elevated circulating IL-6 has been linked to worse motor and cognitive outcomes in multiple cohorts [19], and recent work from the DeNoPa study found correlations between plasma IL-6 and motor impairment in early disease. The concurrent contributions of TNFRSF1A and ICAM-1 implicate tumor-necrosis-factor signaling and endothelial activation, reinforcing the notion that systemic immune dysregulation participates in motor decline. Mechanistically, IL-6 may act through trans-signaling via the soluble IL-6 receptor, promoting microglial activation and exacerbating dopaminergic neurodegeneration in the substantia nigra. Recent Mendelian randomization analyses have provided evidence supporting a causal relationship between genetically determined IL-6 levels and PD risk, strengthening the case that IL-6 is not merely a bystander but an active participant in disease progression.

An important question is whether plasma IL-6 reflects central nervous system inflammation or merely peripheral immune activation. Emerging evidence suggests bidirectional communication between peripheral and central compartments: circulating cytokines can traverse a compromised blood–brain barrier (BBB) in PD [20,21], and, conversely, neuroinflammatory signals may propagate outward via the vagal afferent pathway [22]. The BBB dysfunction observed in PD—particularly in the striatum and substantia nigra—may permit a greater equilibration of inflammatory mediators between compartments, making plasma IL-6 a plausible surrogate for the neuroinflammatory burden. Nevertheless, future studies incorporating paired CSF and plasma sampling would be valuable to clarify this relationship.

The inverse relationship between the BDNF levels and progression risk observed in our model is biologically plausible given the well-documented role of BDNF in supporting dopaminergic neuron survival and maintaining synaptic function [23]. BDNF signals primarily through the TrkB receptor tyrosine kinase, activating downstream pathways (PI3K/Akt, MAPK/ERK) that promote neuronal survival and plasticity. In PD, BDNF expression is reduced in the substantia nigra and striatum at the neuropathological level, and this deficit may compromise compensatory mechanisms that buffer against progressive dopaminergic loss. A lower serum BDNF has been associated with greater motor impairment and cognitive decline in PD [24]; our findings extend this literature by showing that the baseline BDNF has prognostic value for the future motor trajectory. Notably, BDNF is increasingly recognized as a multidimensional biomarker in PD, with studies linking reduced levels not only to motor deficits but also to non-motor manifestations including depression [25] and cognitive impairment [26]. This convergence suggests that BDNF may index a broader neurodegenerative or compensatory capacity that transcends motor circuitry. The appearance of GDNF among the top predictors is particularly intriguing given the ongoing clinical trials of GDNF-based therapies [27], raising the possibility that the baseline neurotrophic tone could inform patient selection for such interventions. Exercise-induced BDNF elevation has been consistently reported in PD patients, suggesting that BDNF may also serve as a modifiable therapeutic target and a biomarker for treatment response monitoring.

Vascular proteins—VEGF-A and HGF—also emerged as key contributors, consistent with the emerging data linking blood–brain barrier dysfunction and cerebral small-vessel disease to neurodegeneration [20,21]. The non-monotonic SHAP profile for VEGF-A suggests that both inadequate and excessive angiogenic signaling may be deleterious, perhaps reflecting VEGF’s dual roles in neuroprotection and vascular permeability. At physiological levels, VEGF-A promotes endothelial survival and neurovascular coupling; however, pathological overexpression increases BBB permeability, facilitating the infiltration of peripheral immune cells and inflammatory mediators into the brain parenchyma. The inflection in the SHAP dependence plot may capture this transition from compensatory angiogenesis to pathological vascular leakage, which is particularly relevant in the nigrostriatal pathway where BBB breakdown is an early feature of PD [20].

As a preliminary exploration of longitudinal protein dynamics, we examined trajectories for the top-3 proteins in the subset of participants with available follow-up Olink measurements (n = 143, with 2–3 time points). IL-6 showed a significantly greater annualized increase in rapid progressors compared to slow progressors (mean ΔNPX/year: 0.18 vs. 0.06; *p* = 0.014), suggesting that IL-6 may function as a dynamic biomarker tracking disease progression rather than as merely a static baseline risk factor. BDNF showed a trend toward a greater decline in rapid progressors (−0.12 vs. −0.05; *p* = 0.068), while VEGF-A trajectories did not differ significantly between groups. These exploratory findings support the biological plausibility of IL-6 as a progression-associated marker, though a comprehensive longitudinal analysis incorporating time-varying covariates and joint modeling is beyond the scope of the current baseline-focused study.

An important consideration, raised during the review, is whether the identified targeted protein signature reflects a PD-specific pathophysiology or accelerated systemic aging, given that IL-6, BDNF, and VEGF-A all change with normal aging. We note that our study objective is prognostic stratification within PD patients rather than PD diagnosis. Age is included as a covariate in the combined model, and the targeted protein model (AUC 0.751) substantially outperforms the clinical model containing age (AUC 0.666), demonstrating that the targeted protein features carry information beyond what age alone explains. Furthermore, a SHAP interaction analysis confirmed that IL-6’s prognostic contribution persists across the age strata. To address this question directly, we compared protein levels in our PD cohort with 97 PPMI healthy controls (see Results). Three proteins—WAS, GDNF, and NEFL—remained significantly elevated in PD after age adjustment (ANCOVA *p* ≤ 0.013), and five proteins demonstrated a monotonic HC → slow PD → rapid PD gradient (all *p* < 0.001), providing direct evidence that these protein changes are proportional to disease severity rather than attributable to aging alone. IL-6, while not significantly elevated in PD versus HC, showed a within-PD gradient consistent with its role as a progression-specific marker.

These findings carry implications for clinical practice in at least two respects. First, a motor progression risk stratification at diagnosis would allow clinicians to tailor monitoring intensity and treatment aggressiveness to the individual prognosis. Second, enriching clinical trials with predicted rapid progressors could shrink the required sample sizes and shorten study timelines, accelerating therapeutic development [28]. The favorable decision-curve profile (Figure 1D) indicates that the model would confer a net benefit across a broad, clinically relevant range of decision thresholds.

For prospective deployment, our model requires Olink PEA measurements from the same four panels used in this study. The trained model, preprocessing pipeline, and prediction interface are provided in the accompanying code repository (see Code Availability). Users applying the model to new datasets should verify that their Olink assay protocol matches PPMI specifications (plate normalization and bridge sample calibration) to ensure NPX value comparability. The prediction module accepts new Olink NPX data and outputs the predicted progression class alongside SHAP-based explanations for individual patients, enabling transparent clinical decision support.

Our comparison of multiple algorithms spanning the interpretability spectrum—from fully transparent models (Decision Tree and Logistic Regression) through semi-transparent models (Elastic Net) to complex ensembles (Random Forest and Gradient Boosting)—revealed a consistent performance–transparency trade-off: fully interpretable models sacrificed approximately 8–12% AUC relative to Random Forest. We argue that a SHAP analysis of the Random Forest achieves an effective compromise: the ensemble’s superior discrimination is preserved while post hoc interpretability is provided at both the global and individual-prediction levels. This is particularly relevant for clinical deployment scenarios where both accuracy and explainability are required for regulatory acceptance and clinician trust.

A methodological consideration concerns the interpretation of SHAP values. We employed TreeSHAP, which—unlike the model-agnostic Kernel SHAP approximation—does not assume feature independence but instead computes exact Shapley values by leveraging the tree structure, naturally accounting for feature dependencies as encoded in the tree splits [13,29]. Nonetheless, while TreeSHAP captures pairwise interactions (as quantified in our interaction analysis), it may not fully capture the higher-order combinatorial effects among biological markers. Cytokines, neurotrophins, and vascular mediators operate within complex signaling networks involving feedback loops and pathway crosstalk that may not be adequately modeled by pairwise interaction terms alone. Future studies employing higher-order interaction methods, pathway-level enrichment analyses, or graph-based approaches may reveal additional combinatorial biomarker signatures that further improve prediction.

The feature-to-sample ratio in our study (~1:7 for 30 features and 211 patients) warrants consideration. While this ratio would be concerning for parametric models, tree-based ensembles are inherently more resistant to overfitting through bagging (Random Forest) and boosting regularization. Importantly, post hoc feature selection analyses using LASSO and recursive feature elimination confirmed that reduced feature sets yielded a comparable model performance, and the top-ranked proteins were consistently retained across all selection methods. The convergent findings from nested cross-validation (AUC 0.743), 100-iteration repeated CV (mean AUC 0.769), and leave-one-site-out analysis (AUC range 0.71–0.79) collectively argue against gross overfitting, though we acknowledge that these internal validation strategies cannot substitute for true external replication.

Although our cohort was predominantly male (63%), the subgroup analyses demonstrated a comparable model performance across sexes and age groups, with no clustering of prediction errors within any demographic subgroup. Prior work has highlighted the importance of demographic factors in predictive modeling, and we acknowledge that comprehensive fairness audits across ethnicity, socioeconomic status, and clinical site should be conducted in larger, more diverse validation cohorts to ensure equitable model performance before clinical deployment.

A potential concern is whether dopaminergic medication influences the plasma proteome. Although a subset of our cohort had initiated therapy by the time of blood collection, a sensitivity analysis restricted to treatment-naïve patients yielded similar discrimination and identical top-ranked proteins. Additionally, the inclusion of LEDD as a continuous covariate had a negligible impact on the model performance or feature rankings. Prior studies have reported variable effects of levodopa on peripheral inflammatory markers [30] and neurotrophins [31]; however, the low doses and short treatment durations in our early-stage cohort may have minimized such confounding. Nonetheless, we cannot exclude subtle medication effects, and future longitudinal studies with serial sampling before and after treatment initiation would be informative.

This study has limitations. The single-cohort design without external validation means that the generalizability to other populations remains unconfirmed; we emphasize that external validation in independent cohorts (PDBP, DeNoPa, and LRRK2 Cohort Consortium) is essential before clinical deployment, and the current results should be viewed as hypothesis-generating. PPMI enrolls predominantly early-stage patients at specialized centers, which may limit the applicability to community settings or more advanced disease. The binary outcome, while pragmatic, collapses a continuous spectrum of progression phenotypes; however, sensitivity analyses using extreme quartiles and a clinically anchored threshold (≥5 points/year) demonstrated even stronger discrimination, supporting the biological meaningfulness of the dichotomy. The concordance between the median-split, quartile, clinically anchored, and continuous outcome analyses supports the robustness of the identified biomarkers; for clinical implementation, clinically anchored thresholds would be preferred to avoid the population-specificity inherent in median splits. Only 28 proteins from four targeted Olink panels were analyzed, from an initial set of 276 measured analytes (Supplementary Table S10); broader platforms—such as the SomaScan 7000-plex or unbiased mass spectrometry approaches—might uncover additional markers and provide a more comprehensive pathway coverage. Importantly, our healthy control comparison, while demonstrating PD-specific elevation for several proteins (three surviving age adjustment via ANCOVA), is limited by the 7.2-year age difference between the PD and HC groups; prospective studies with strictly age-matched controls would provide more definitive evidence regarding the identified signatures to PD-specific mechanisms versus accelerated systemic aging. Nevertheless, the age-adjusted analyses (ANCOVA), the monotonic HC→slow→rapid gradient, and the substantial outperformance of clinical models by models and the convergence with PD-specific mechanistic literature support biological specificity. Our exclusive focus on motor progression is a further limitation: non-motor symptoms—particularly cognitive impairment, depression, and autonomic dysfunction—are often more burdensome for patients and may have different protein correlates. The extension to multimodal clinical outcomes (motor + cognitive + psychiatric + autonomic composite endpoints) is a priority for future work. Finally, cross-sectional protein measurements cannot capture dynamic changes in protein expression that might track—or even predict—clinical inflection points, although our preliminary longitudinal analysis of IL-6 trajectories provides initial support for such dynamics.

The next steps should include validation in independent cohorts such as PDBP and DeNoPa, the extension to continuous and non-motor outcomes—particularly cognitive decline and dementia prediction, which would carry immeasurably greater clinical value—integration with genetic and imaging biomarkers, and longitudinal protein sampling to characterize the temporal dynamics. A longitudinal analysis of serial Olink measurements—examining whether protein trajectory changes precede, parallel, or follow motor inflection points—would be highly informative for understanding the temporal dynamics of the identified biomarkers and could enable dynamic risk updating. The healthy control comparison presented herein provides initial evidence for the PD specificity of several protein markers; further validation with larger, strictly age-matched control cohorts would strengthen the conclusions regarding aging-related changes. Methodological advances in data balancing and generative AI for small clinical datasets [16] may offer additional strategies for improving model robustness and generalizability. The development of streamlined, point-of-care assays for the top-ranked proteins would bring the approach closer to clinical implementation.

## 4. Conclusions

This study demonstrates that targeted plasma protein profiling, when analyzed using interpretable machine-learning methods, provides superior risk stratification for motor progression in early PD compared with conventional clinical predictors alone. SHAP-derived feature attributions implicate inflammatory (IL-6 and TNFRSF1A), neurotrophic (BDNF and GDNF), and vascular (VEGF-A and HGF) pathways—findings that are biologically coherent and may inform therapeutic targeting. Feature importance rankings were highly stable across cross-validation folds, multiple outcome definitions, and leave-one-site-out validation. Sensitivity analyses confirmed the robustness of the classification approach, and decision curve analysis demonstrated clinical utility across a practical range of thresholds. Validation in external cohorts is warranted before clinical adoption.

## 5. Methods

### 5.1. Study Population

Data were drawn from the PPMI database (www.ppmi-info.org), an ongoing multicenter observational study sponsored by The Michael J. Fox Foundation that enrolls participants at 33 sites worldwide [32,33]. We included patients with a clinical PD diagnosis meeting UK Brain Bank criteria, confirmed by abnormal dopamine-transporter imaging, who had (i) baseline Olink plasma protein data, (ii) at least 2 years of motor follow-up, and (iii) complete demographic and clinical covariates. Patients with atypical or secondary parkinsonism, or with >20% missing protein values, were excluded. The PPMI protocol was approved by the institutional review board at each of the 33 participating sites under the oversight of the University of Rochester (Rochester, NY, USA) as the coordinating center (PPMI ClinicalTrials.gov identifier: NCT01141023); all participants gave written informed consent prior to data collection.

In this study, “baseline” refers to the PPMI enrolment visit, at which time plasma samples were collected for protein analysis. Although PPMI targets de novo, treatment-naïve PD patients, a subset of participants had initiated low-dose dopaminergic therapy by the time of their baseline blood draw due to clinical necessity or slight delays between diagnosis and enrolment. Medication status at baseline was documented, and levodopa-equivalent daily dose (LEDD) was calculated using standard conversion factors. For the primary analysis, we included all patients regardless of medication status to maximize statistical power; sensitivity analyses stratified by treatment status were conducted to assess potential confounding (see Statistical analysis).

### 5.2. Plasma Protein Measurements

Baseline plasma was assayed with Olink PEA technology across four targeted panels, Cardiovascular II (CARDIO), Neurology (NEURO), Inflammation (INF), and Oncology II (ONC), which collectively measured 276 analytes. PEA uses paired antibody-conjugated oligonucleotide probes whose hybridization generates a unique DNA amplicon, enabling highly multiplexed measurement from minimal sample volume [7]. This represents a targeted proteomic approach rather than comprehensive proteome-wide profiling. Following quality control—applied in a stepwise fashion—proteins were excluded for: (a) >50% below-detection values (n = 89), indicating insufficient assay sensitivity; (b) coefficient of variation > 30% across replicate samples (n = 34), indicating poor technical reproducibility; (c) >20% sample-level missingness (n = 68), a conservative threshold to ensure imputation reliability; and (d) interquartile range ≤ 0.5 NPX units (n = 57), indicating minimal biological variability. The complete list of all 276 measured proteins, with per-protein missingness rate, below-LOD rate, coefficient of variation, interquartile range, and specific exclusion criterion, is provided in Supplementary Table S10. The 28 analytes passing all four criteria were retained for analysis. Per-protein missingness ranged from 0% to 18.4%, with no significant differences between progression groups (Supplementary Table S1). The median below-limit-of-detection rate was 3.8% (range 0–12.1%). The median number of missing values per patient was 1 (IQR 0–3); of the total 5,908 measurements (211 × 28), 284 values (4.8%) required imputation. Missing values were imputed via k-nearest neighbors (k = 5). The choice of k = 5 was based on a systematic evaluation of k values (3, 5, 7, and 10) using normalized root-mean-square error (NRMSE) from a 20% artificial missingness experiment; k = 5 yielded the lowest NRMSE (0.082), consistent with published recommendations for targeted protein datasets of comparable dimensionality. Sensitivity analyses using LOD/√2 substitution and multivariate imputation by chained equations (MICE) yielded concordant model performance (AUC range: 0.745–0.758; Supplementary Material). All NPX values are log2-scaled, with higher values denoting higher concentrations.

### 5.3. Outcome Definition

Motor progression was quantified as the annualized change in MDS-UPDRS Part III score, assessed in the practically defined OFF state (≥6 h after last dopaminergic dose) by certified raters [34,35]. Patients whose annual change exceeded the cohort median were designated “rapid progressors”; the remainder were labeled “slow progressors.” This dichotomization, though simplified, facilitates clinical interpretation and comparability with prior prognostic studies. To address the population-specificity of this threshold, we also evaluated a clinically anchored definition (MDS-UPDRS Part III increase ≥ 5 points/year, approximating the minimal clinically important difference) and continuous progression modeling as sensitivity analyses.

### 5.4. Machine-Learning Models

The full training pipeline was implemented as follows for every model evaluation run. First, the dataset was split into training and test folds using 5-fold stratified cross-validation, preserving the class distribution in each fold. Within each training fold, (i) missing values were imputed using k-nearest neighbors (k = 5) [36] fitted exclusively on training data; (ii) features were z-score standardized using the mean and standard deviation computed from training data only; and (iii) the classifier was trained. The stored imputer and scaler were then applied to the held-out test fold before prediction, ensuring strict separation of training and test data and preventing any data leakage.

Three primary classifiers were evaluated: L2-regularised Logistic Regression (C = 1.0) as a linear baseline, Random Forest [37] (100 trees, maximum depth 5), and Gradient Boosting (100 estimators, maximum depth 3). Hyperparameters were selected through a preliminary nested 3-fold cross-validation grid search on 70% of the data prior to the main evaluation. Grid ranges explored included n_estimators ∈ {50, 100, 200}, and max_depth ∈ {3, 5, 7, None} for Random Forest, and learning_rate ∈ {0.01, 0.1, 0.3}, and max_depth ∈ {2, 3, 5} for Gradient Boosting. The selected hyperparameters were then fixed for all subsequent 5-fold CV runs to avoid optimistic bias. Additionally, a single Decision Tree classifier and Elastic Net (L1 ratio = 0.5) were included to represent fully transparent models across the interpretability spectrum. We examined three input configurations: targeted-protein-only (28 proteins), clinical-only (age, baseline MDS-UPDRS III), and combined. Analyses used Python 3.10 and scikit-learn 1.3.0.

### 5.5. Model Evaluation

Performance was assessed via 5-fold stratified cross-validation. The primary metric was AUC-ROC, with 95% confidence intervals from 1000 bootstrap resamples. Secondary metrics—sensitivity, specificity, positive and negative predictive values, and F1-score—were computed at the threshold maximizing Youden’s J. Model calibration was judged by Brier score and graphical inspection of calibration curves. Clinical utility was appraised through decision curve analysis, which plots net benefit against threshold probability and compares the model to default “treat all” and “treat none” strategies [38].

To assess the stability of cross-validation estimates, we repeated the entire 5-fold cross-validation procedure 100 times with different random seeds and reported the distribution of AUC values. To provide a less optimistically biased performance estimate, we additionally performed nested cross-validation (outer 5-fold and inner 3-fold for hyperparameter selection). This repeated cross-validation approach provides a more comprehensive assessment of model variability than a single cross-validation run.

### 5.6. SHAP Analysis

SHAP values were computed with TreeExplainer, which yields exact Shapley values for tree ensembles in polynomial time [13,29]. Unlike the model-agnostic Kernel SHAP approximation, TreeSHAP does not assume feature independence; it accounts for feature dependencies as encoded in the tree structure, making it appropriate for correlated biological markers [13]. A positive SHAP value for a given protein in a given patient indicates that the protein level pushed the prediction toward rapid progression; negative values indicate the opposite. Global importance was summarized as mean |SHAP| across all patients ± SD across the 500 models (5 folds × 100 iterations), with bootstrap 95% confidence intervals. Beeswarm and dependence plots were generated to visualize feature-outcome relationships. Feature ranking stability was assessed by computing Spearman rank correlations between fold-level SHAP rankings. SHAP interaction values were computed using TreeSHAP to quantify pairwise feature interactions. Analyses used the shap package (v 0.42.1) [14].

### 5.7. Healthy Control Comparison

To assess the PD specificity of the identified protein signatures, we compared baseline Olink plasma protein levels in PD patients with PPMI healthy control participants (n = 97) who had concurrent measurements from the same four panels. Demographic characteristics were compared using Welch’s *t*-test (continuous) and χ^2^ test (categorical). Protein-level comparisons employed Welch’s *t*-tests with Benjamini–Hochberg FDR correction (q < 0.05). To address the age difference between PD and HC groups, ANCOVA was performed with age as covariate. A three-group gradient analysis (HC vs. slow progressors vs. rapid progressors) was conducted using one-way ANOVA to test for monotonic trends in protein levels across disease severity strata. Missing values in the HC cohort were imputed using the same k-nearest neighbors procedure (k = 5) applied to the PD cohort. All HC comparison analyses were performed post hoc in response to reviewer request.

### 5.8. Statistical Analysis

Baseline characteristics were compared between progression groups with *t*-tests (continuous) or χ^2^ tests (categorical). AUC differences were tested via DeLong’s method. All tests were two-sided; *p* < 0.05 was considered significant. Statistical routines came from SciPy 1.11.0 and statsmodels 0.14.0.

Two pre-specified sensitivity analyses were conducted. First, to assess the robustness of the median-split dichotomization, we repeated the analysis comparing only extreme quartiles: the fastest 25% of progressors versus the slowest 25%. Second, to evaluate potential confounding by baseline medication, we restricted the analysis to treatment-naïve patients and compared model performance and feature rankings to the full cohort. Additional post hoc sensitivity analyses included the following: (i) a clinically anchored outcome definition (MDS-UPDRS Part III increase ≥ 5 points/year); (ii) continuous outcome modeling using Random Forest regression; (iii) LEDD as a continuous covariate; (iv) leave-one-site-out cross-validation for the five largest PPMI sites; (v) subgroup analyses by sex and age group; (vi) feature selection using LASSO and recursive feature elimination; (vii) alternative imputation methods (LOD/√2 and MICE); and (viii) relaxed protein filtering threshold (missingness ≤ 30%, retaining 35 proteins). Results are presented in the Supplementary Materials.

## Figures and Tables

**Table 1:** Baseline demographic and clinical characteristics of the study cohort. Patients were classified as rapid or slow progressors based on the median annualized change in MDS-UPDRS Part III score (2.1 points/year). Continuous variables are presented as mean ± SD or median [interquartile range]; categorical variables as n (%). *p* values were calculated using independent-samples *t*-test (continuous) or χ^2^ test (categorical).

Variable	Total (n = 211)	Rapid Progressors (n = 105)	Slow Progressors (n = 106)	*p* Value
***Demographics***				
Age, years (mean ± SD)	61.8 ± 10.2	63.4 ± 9.8	60.2 ± 10.4	0.022 *
Male sex, n (%)	133 (63.0)	68 (64.8)	65 (61.3)	0.604
***Clinical Characteristics***				
Disease duration, years (mean ± SD)	1.8 ± 1.1	1.9 ± 1.2	1.7 ± 1.0	0.218
Baseline MDS-UPDRS III (mean ± SD)	20.4 ± 9.3	22.8 ± 9.6	18.1 ± 8.5	<0.001 **
Annual motor change, pts/yr (median [IQR])	2.1 [0.8–3.9]	3.9 [2.8–5.4]	0.8 [0.2–1.4]	<0.001 **
Follow-up, years (median [IQR])	4.2 [3.1–5.4]	4.1 [3.0–5.2]	4.3 [3.2–5.5]	0.487
***Treatment Status at Baseline***				
Treatment-naïve, n (%)	127 (60.2)	61 (58.1)	66 (62.3)	0.418
LEDD among treated, mg/day (mean ± SD)	354 ± 142	362 ± 148	345 ± 135	0.561

* *p* < 0.05; ** *p* < 0.01. MDS-UPDRS, Movement Disorder Society–Unified Parkinson’s Disease Rating Scale; LEDD, levodopa-equivalent daily dose; IQR, interquartile range; SD, standard deviation.

## Data Availability

The data presented in this study are openly available from the Parkinson’s Progression Markers Initiative (PPMI) database at www.ppmi-info.org. PPMI is a public-private partnership funded by The Michael J. Fox Foundation for Parkinson’s Research. Access requires free registration and acceptance of the PPMI data use agreement. A derived dataset containing the 28 retained protein NPX values (before imputation), clinical covariates, outcome variables, and per-protein missingness indicators has been deposited on Zenodo (DOI: [to be assigned upon acceptance]) in compliance with FAIR data sharing principles. The analysis code is available at https://github.com/ [repository] and has been archived on Zenodo (DOI: [to be assigned upon acceptance]). The repository includes a reproducible pipeline with modular scripts for data preprocessing, KNN imputation, model training and evaluation, SHAP analysis, and all sensitivity analyses, together with a prediction module that accepts new Olink NPX data and outputs the predicted progression class with SHAP-based individual explanations. A comprehensive README provides step-by-step instructions for reproducing all figures, tables, and supplementary analyses.

## Supplementary Materials

The following supporting information can be downloaded at: https://www.mdpi.com/article/10.3390/bioengineering13040380/s1.
