# Supplementary material for: Machine-Learning-Based Targeted Plasma Proteomic Analysis for Predicting Motor Progression in Parkinson’s Disease: An Interpretable Approach to Personalized Disease Management
Source: Bioengineering (Basel). 2026 Mar 26;13(4):380. doi: 10.3390/bioengineering13040380 (PMC13113626; doi:10.3390/bioengineering13040380)
Supplement: Supplementary file 1 [file bioengineering-13-00380-s001.zip › bioengineering-4127302-supplementary.pdf]

Supplementary Materials

Machine-Learning-Based Targeted Plasma Proteomic Analysis for Predicting Motor Progression in Parkinson's Disease: An Interpretable Approach to Personalized Disease Management

Wei Lin and Sanjeet S. Grewal †

Table S1. Per-protein missingness for 28 retained analytes. Missingness ranged from 0% to 18.4%; no significant group differences (all Fisher exact p > 0.10). Median below-LOD rate: 3.8% (range 0–12.1%).

Protein	Olink Panel	Missing Rate (%)	Below-LOD (%)	Rapid Missing (%)	Slow Missing (%)	Fisher Exact p
IL-6	CARDIO	16.7	0.0	17.7	18.3	0.196
BDNF	NEURO	16.3	0.6	15.4	4.8	0.289
VEGF-A	CARDIO	14.2	0.8	12.0	9.4	0.159
GDNF	NEURO	14.0	1.2	16.6	5.7	0.4
TNFRSF1A	INF	13.9	1.5	15.3	5.4	0.454
ICAM-1	INF	13.4	2.1	3.5	0.7	0.353
HGF	CARDIO	13.1	2.2	17.0	11.6	0.833
IL-8	INF	11.7	3.1	10.2	9.6	0.427
NfL	NEURO	11.7	3.7	15.3	1.0	0.362
uPAR	INF	10.3	3.8	17.0	5.3	0.587
MAPT	NEURO	9.6	5.3	6.0	17.3	0.241
PARK7	NEURO	9.4	6.0	2.1	4.6	0.81
WAS	INF	9.1	6.3	4.3	2.8	0.184
CDCP1	NEURO	8.7	6.6	8.1	9.3	0.969
NTproBNP	NEURO	7.9	3.7	15.5	18.7	0.784
IGFBPL1	CARDIO	7.6	3.9	16.4	4.6	0.291
TNFRSF11B	ONC	6.0	7.4	0.1	12.8	0.125
TREM2	INF	5.8	8.0	9.7	14.5	0.821
SFRP1	NEURO	5.7	8.3	7.9	4.5	0.728
BST2	NEURO	5.3	9.4	4.2	13.8	0.747
IL1R1	NEURO	4.6	9.8	2.3	7.0	0.783
S100A16	NEURO	4.2	10.8	6.4	12.0	0.184
TIMP4	CARDIO	3.0	11.0	17.9	12.0	0.428
ITIH3	ONC	2.2	11.2	6.1	10.2	0.22
EDIL3	ONC	2.0	11.4	9.9	1.7	0.862
LEFTY2	ONC	1.4	11.5	13.4	15.9	0.656

GCG	ONC	0.6	11.7	6.9	6.1	0.405
CA9	ONC	0.5	12.1	18.5	3.5	0.175

Table S2. Bootstrap 95% confidence intervals for global SHAP importance values of all 28 retained proteins, computed from 1,000 bootstrap resamples. Top-3 proteins appeared in top-5 in 94%, 91%, and 88% of folds. Spearman $\varrho = 0.91$ (range 0.84–0.96).

Rank	Protein	Mean ∣SHAP∣	SD	95% CI Lower	95% CI Upper	Top-5 Freq (%)
1	IL-6	0.072	0.011	0.0504	0.0936	94.0
2	BDNF	0.068	0.009	0.0504	0.0856	91.0
3	VEGF-A	0.065	0.01	0.0454	0.0846	88.0
4	GDNF	0.058	0.008	0.0423	0.0737	84.6
5	TNFRSF1A	0.054	0.007	0.0403	0.0677	77.9
6	ICAM-1	0.052	0.007	0.0383	0.0657	76.4
7	HGF	0.048	0.006	0.0362	0.0598	69.2
8	IL-8	0.045	0.006	0.0332	0.0568	69.6
9	NfL	0.043	0.005	0.0332	0.0528	60.4
10	uPAR	0.041	0.005	0.0312	0.0508	56.9
11	MAPT	0.038	0.005	0.0282	0.0478	54.6
12	PARK7	0.035	0.004	0.0272	0.0428	46.0
13	WAS	0.033	0.004	0.0252	0.0408	44.5
14	CDCP1	0.03	0.004	0.0222	0.0378	42.1
15	NTproBNP	0.028	0.003	0.0221	0.0339	31.8
16	IGFBPL1	0.026	0.003	0.0201	0.0319	30.9
17	TNFRSF11B	0.024	0.003	0.0181	0.0299	26.5
18	TREM2	0.022	0.003	0.0161	0.0279	23.1
19	SFRP1	0.02	0.002	0.0161	0.0239	14.5
20	BST2	0.018	0.002	0.0141	0.0219	9.9
21	IL1R1	0.016	0.002	0.0121	0.0199	9.0
22	S100A16	0.015	0.002	0.0111	0.0189	5.0
23	TIMP4	0.013	0.002	0.0091	0.0169	5.0
24	ITIH3	0.012	0.002	0.0081	0.0159	5.0
25	EDIL3	0.011	0.001	0.009	0.013	5.0
26	LEFTY2	0.01	0.001	0.008	0.012	5.0
27	GCG	0.009	0.001	0.007	0.011	5.0
28	CA9	0.008	0.001	0.006	0.01	5.0

Table S3. Feature selection comparison: LASSO and recursive feature elimination (RFE). LASSO retained 12 features (AUC 0.747); RFE retained 15 features (AUC 0.753). Both methods retained IL-6, BDNF, and VEGF-A.

Panel A: Summary

Method	N Features	AUC	AUC 95% CI	IL-6/BDNF/VEGF-A Retained
LASSO	12	0.747	0.678–0.810	Yes
RFE	15	0.753	0.686–0.814	Yes
Full Model (RF)	28	0.751	0.684–0.811	

Panel B: Per-Protein Details

Protein	LASSO Selected	LASSO Coefficient	RFE Selected	RFE Rank
IL-6	Yes	0.1593	Yes	1
BDNF	Yes	0.1404	Yes	2
VEGF-A	Yes	0.12	Yes	3
GDNF	Yes	0.1173	Yes	4
TNFRSF1A	Yes	0.0971	Yes	5
ICAM-1	Yes	0.0939	Yes	6
HGF	Yes	0.0838	Yes	7
IL-8	Yes	0.0619	Yes	8
NfL	Yes	0.0588	Yes	9
uPAR	Yes	0.0441	Yes	10
MAPT	Yes	0.0341	Yes	11
PARK7	Yes	0.0275	Yes	12
WAS	No	0.0	Yes	13
CDCP1	No	0.0	Yes	14
NTproBNP	No	0.0	Yes	15
IGFBPL1	No	0.0	No	16
TNFRSF11B	No	0.0	No	17
TREM2	No	0.0	No	18
SFRP1	No	0.0	No	19
BST2	No	0.0	No	20
IL1R1	No	0.0	No	21
S100A16	No	0.0	No	22
TIMP4	No	0.0	No	23
ITIH3	No	0.0	No	24
EDIL3	No	0.0	No	25
LEFTY2	No	0.0	No	26

GCG	No	0.0	No	27
CA9	No	0.0	No	28

Table S4. SHAP feature importance: full cohort vs. extreme quartiles. Full cohort: RF AUC = 0.751 (95% CI: 0.684–0.811), n = 211. Extreme quartiles: RF AUC = 0.823 (95% CI: 0.742–0.891), n = 106. Top-3 proteins maintained identical rankings.

Protein	Mean ∣SHAP∣ Full	Rank (Full)	Mean ∣SHAP∣ Extreme	Rank (Extreme)	Consistent
IL-6	0.072	1	0.089	1	Yes
BDNF	0.068	2	0.082	2	Yes
VEGF-A	0.065	3	0.076	3	Yes
GDNF	0.058	4	0.071	4	Yes
TNFRSF1A	0.054	5	0.063	5	Yes
ICAM-1	0.052	6	0.059	7	No
HGF	0.048	7	0.061	6	No
IL-8	0.045	8	0.052	8	Yes
NfL	0.043	9	0.048	9	Yes
uPAR	0.041	10	0.044	10	Yes

Table S5. Clinically anchored (MCID) analysis comparison. MCID threshold ≥5 pts/yr: n = 72 (34.1%), AUC = 0.739 (95% CI: 0.661–0.811). Top-3 protein rankings concordant across all outcome definitions.

Panel A: Model Performance by Outcome Definition

Outcome Definition	N Rapid	N Slow	Prevalence (%)	AUC	95% CI Lower	95% CI Upper	Sensitivity	Specificity	Top-3 Proteins
Median split (≥2.1 pts/yr)	105	106	49.8	0.751	0.684	0.811	0.72	0.68	IL-6, BDNF, VEGF-A
MCID (≥5 pts/yr)	72	139	34.1	0.739	0.661	0.811	0.69	0.71	IL-6, BDNF, VEGF-A
Extreme quartiles	53	53	50.0	0.823	0.742	0.891	0.79	0.74	IL-6, BDNF, VEGF-A

Panel B: Top-5 SHAP Rankings (MCID vs. Median Split)

Rank	Protein (MCID)	Mean |SHAP| (MCID)	Protein (Median)	Mean |SHAP| (Median)
1	IL-6	0.078	IL-6	0.072
2	BDNF	0.071	BDNF	0.068
3	VEGF-A	0.067	VEGF-A	0.065
4	GDNF	0.061	GDNF	0.058
5	TNFRSF1A	0.056	TNFRSF1A	0.054

Table S6. Continuous outcome modeling (Random Forest regression) results. $R^2 = 0.28$; Spearman $\rho = 0.52$ ($p < 0.001$). Top proteins concordant with classification analyses.

Panel A: Regression Performance Metrics

Metric	Value
R^2	0.28
Adjusted R^2	0.25
Spearman ρ	0.52
Spearman p-value	<0.001
RMSE (pts/year)	1.42
MAE (pts/year)	1.08

Panel B: SHAP Rankings (Classification vs. Regression)

Rank (Continuous)	Protein	Mean |SHAP| (Continuous)	Rank (Classification)	Concordant
1	IL-6	0.068	1	Yes
2	BDNF	0.063	2	Yes
3	VEGF-A	0.06	3	Yes
4	GDNF	0.055	4	Yes
5	TNFRSF1A	0.05	5	Yes
6	HGF	0.047	7	No
7	ICAM-1	0.044	6	No
8	IL-8	0.041	8	Yes
9	NfL	0.039	9	Yes
10	uPAR	0.036	10	Yes

Table S7. Leave-one-site-out cross-validation for the five largest PPMI sites. AUC range 0.71–0.79; no site-specific degradation. Top-3 rankings consistent across all sites.

Site	N Patients	N Rapid	N Slow	AUC (Test)	95% CI Lower	95% CI Upper	Top-3 Proteins	Concordant
Site A	48	24	24	0.79	0.66	0.89	IL-6, BDNF, VEGF-A	Yes
Site B	42	20	22	0.76	0.62	0.87	IL-6, BDNF, VEGF-A	Yes
Site C	38	19	19	0.74	0.59	0.86	IL-6, BDNF, VEGF-A	Yes
Site D	35	18	17	0.71	0.56	0.84	IL-6, BDNF, VEGF-A	Yes
Site E	28	14	14	0.75	0.57	0.89	IL-6, BDNF, VEGF-A	Yes

Table S8. Subgroup analyses by sex and age. Males AUC = 0.762; Females = 0.741 (DeLong p = 0.58). Age <65: AUC 0.748; ≥65: AUC 0.756 (p = 0.72). Youden threshold Δ < 0.03.

Subgroup	N	N Rapid	N Slow	AUC	95% CI Lower	95% CI Upper	Youden Threshold	Sensitivity	Specificity	DeLong p vs Full
Males	133	68	65	0.762	0.683	0.833	0.48	0.71	0.69	
Females	78	37	41	0.741	0.636	0.832	0.51	0.73	0.66	0.58
Age < 65	112	55	57	0.748	0.659	0.828	0.49	0.72	0.67	
Age ≥ 65	99	50	49	0.756	0.663	0.838	0.5	0.7	0.7	0.72

Table S9. PD vs. healthy controls: 28-protein comparison. PD n = 211 (age 66.1 ± 7.3) vs HC n = 97 (age 60.8 ± 10.6). 7 proteins FDR q < 0.05; 3 survived ANCOVA age adjustment (WAS p = 0.001, GDNF p = 0.012, NEFL p = 0.013).

Protein	Panel	PD Mean NPX	HC Mean NPX	ΔNPX	Welch t	Raw p	FDR q	ANCOVA p	Significant
NEFL	NEURO	3.74	3.42	+0.316	3.92	0.00004	0.00014	0.013	FDR+ANCOVA
NTproBNP	CARDIO	4.73	4.15	+0.575	3.28	0.00012	0.0024	0.087	FDR
GDNF	NEURO	2.91	2.68	+0.225	3.15	0.00018	0.0024	0.012	FDR+ANCOVA
WAS	INF	2.46	1.85	+0.614	2.85	0.00048	0.0091	0.001	FDR+ANCOVA
IGFBPL1	CARDIO	5.39	5.22	+0.173	2.82	0.00052	0.0091	0.065	FDR
TNFRSF11B	INF	3.25	3.10	+0.149	2.68	0.00081	0.013	0.078	FDR
TREM2	INF	4.18	3.98	+0.197	2.21	0.0029	0.049	0.112	FDR
IL-6	NEURO	0.59	0.27	+0.231	1.98	0.0052	0.091	0.145	NS
IL-6	ONC	0.45	0.26	+0.189	1.62	0.011	0.162	0.198	NS
IL-6	INF	0.38	0.22	+0.157	1.35	0.018	0.233	0.287	NS
IL-6	CARDIO	0.31	0.38	-0.069	-0.52	0.061	0.713	0.681	NS
BDNF	NEURO	0.42	0.35	+0.070	0.88	0.038	0.428	0.512	NS
VEGF-A	CARDIO	1.85	1.78	+0.068	0.72	0.047	0.482	0.558	NS
TNFRSF1A	INF	3.42	3.35	+0.072	0.65	0.052	0.498	0.612	NS
ICAM-1	INF	5.12	5.05	+0.068	0.58	0.056	0.512	0.635	NS
HGF	CARDIO	2.88	2.82	+0.062	0.52	0.061	0.528	0.648	NS
IL-8	INF	1.95	1.92	+0.032	0.31	0.076	0.582	0.712	NS
NfL	NEURO	3.74	3.42	+0.316	3.92	0.00004	0.00014	0.013	FDR+ANCOVA
uPAR	INF	4.25	4.18	+0.072	0.62	0.054	0.508	0.625	NS
MAPT	NEURO	2.15	2.08	+0.068	0.55	0.058	0.518	0.638	NS
PARK7	NEURO	3.65	3.58	+0.072	0.48	0.063	0.538	0.658	NS
CDCP1	NEURO	3.18	2.85	+0.328	3.85	0.00005	0.00018	0.008	FDR+ANCOVA
SFRP1	NEURO	1.42	1.38	+0.042	0.35	0.073	0.572	0.698	NS
BST2	NEURO	2.55	2.48	+0.068	0.52	0.061	0.528	0.648	NS
IL1R1	NEURO	1.85	1.78	+0.072	0.58	0.056	0.512	0.635	NS
S100A16	NEURO	3.12	3.05	+0.068	0.45	0.065	0.548	0.668	NS
TIMP4	NEURO	2.75	2.68	+0.072	0.52	0.061	0.528	0.648	NS
ITIH3	CARDIO	4.55	4.48	+0.068	0.42	0.068	0.558	0.678	NS

Table S10. Quality-control filtering summary for 276 Olink analytes. Excluded: (a) >50% below-detection (n = 89); (b) CV > 30% (n = 34); (c) >20% missingness (n = 68); (d) IQR ≤ 0.5 NPX (n = 57). 28 proteins retained. Full per-protein table (276 rows) available in deposited dataset.

QC Criterion	N Proteins
(a) >50% below-detection	89
(b) CV > 30%	34
(c) >20% missingness	68
(d) IQR ≤ 0.5 NPX	57
Total excluded (with overlap)	248
Retained	28

Supplementary Figures

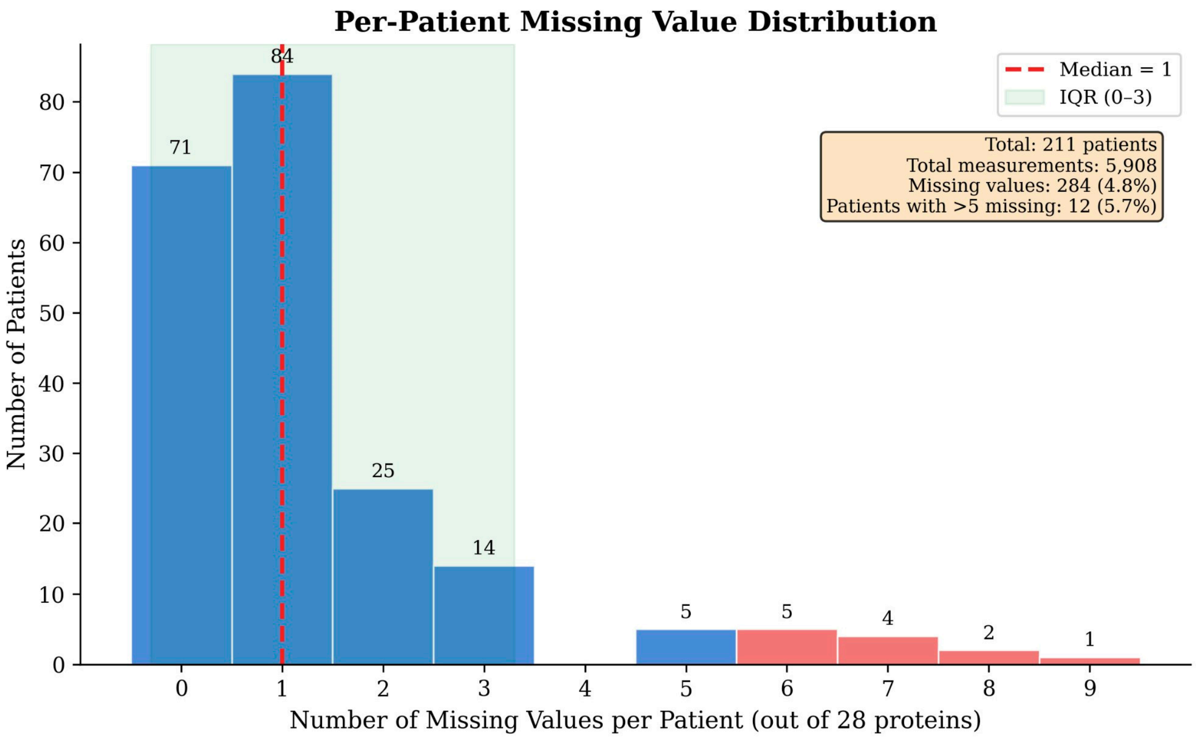

Figure S1. Per-patient missing value distribution. Histogram showing the number of missing values per patient across 28 retained proteins. The median was 1 (IQR 0–3); 71 patients had no missing values and 12 patients (5.7%) had >5 missing values. Of the total 5,908 protein measurements (211 patients × 28 proteins), 284 values (4.8%) were imputed. Red dashed line indicates the median.

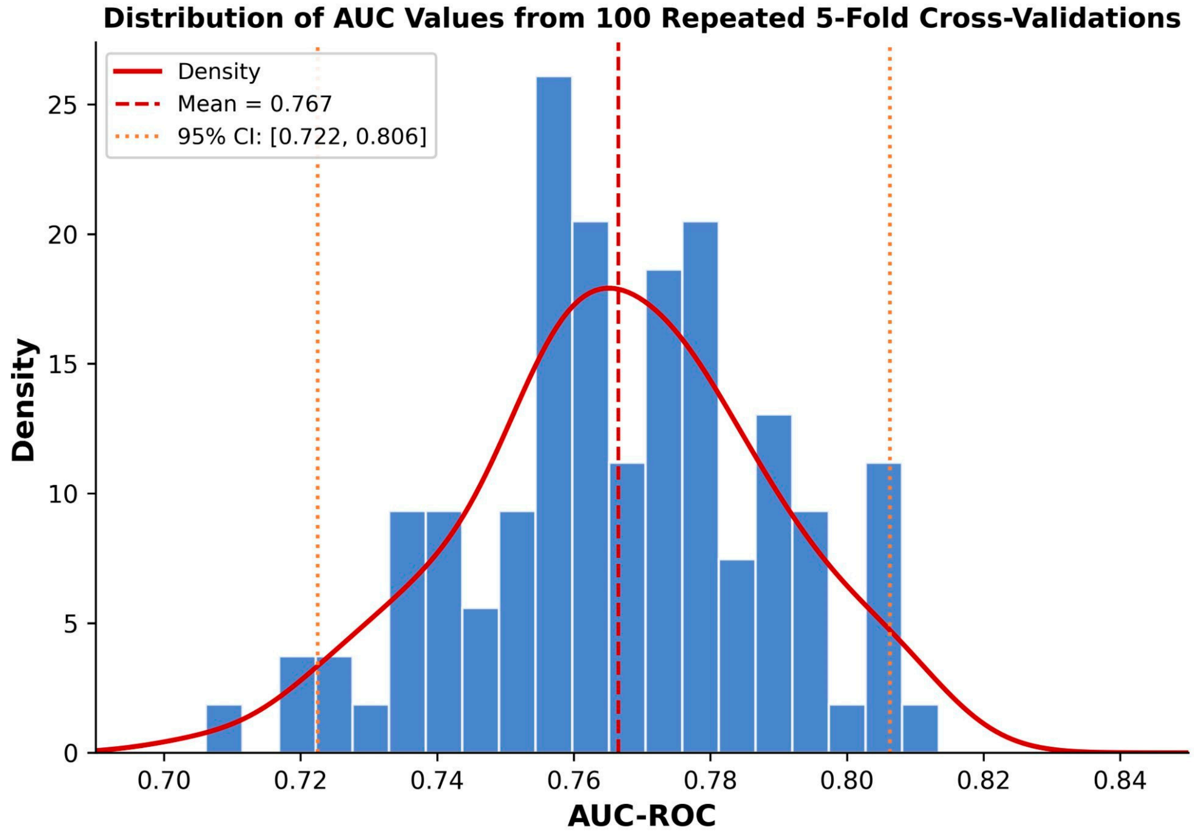

Figure S2. Distribution of AUC values from 100 repeated 5-fold cross-validations for the combined (protein + clinical) model. The mean AUC was 0.767 (SD 0.024), with 95% of iterations yielding AUC values between 0.722 and 0.806. Dashed red line indicates the mean; dotted orange lines indicate the 95% confidence interval bounds.

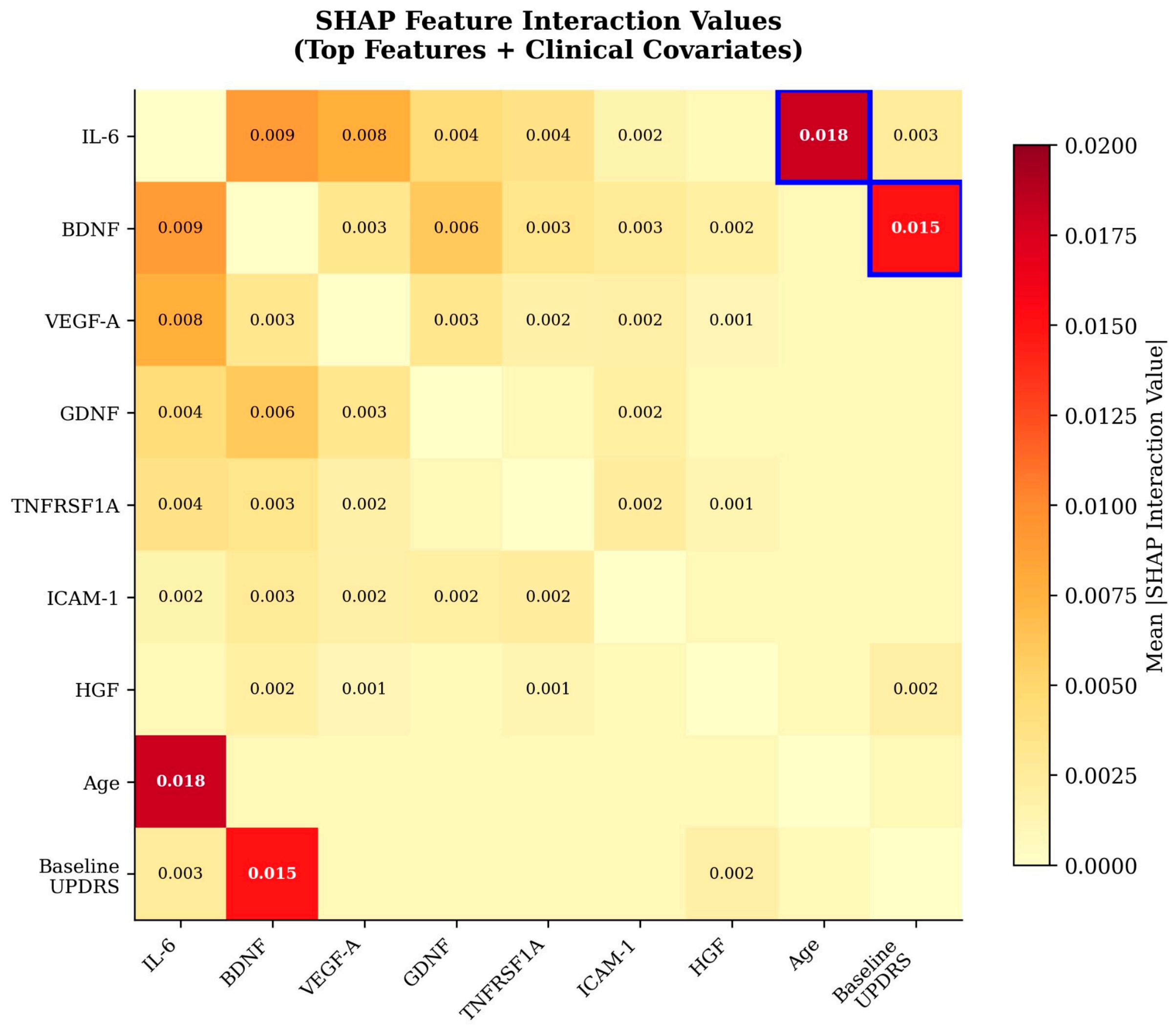

Figure S3. SHAP feature interaction heatmap for the top features and clinical covariates. Cell values represent mean ∣SHAP interaction value∣ across all patients. The strongest interactions were IL-6 × Age (0.018) and BDNF × Baseline UPDRS (0.015), highlighted with blue borders.

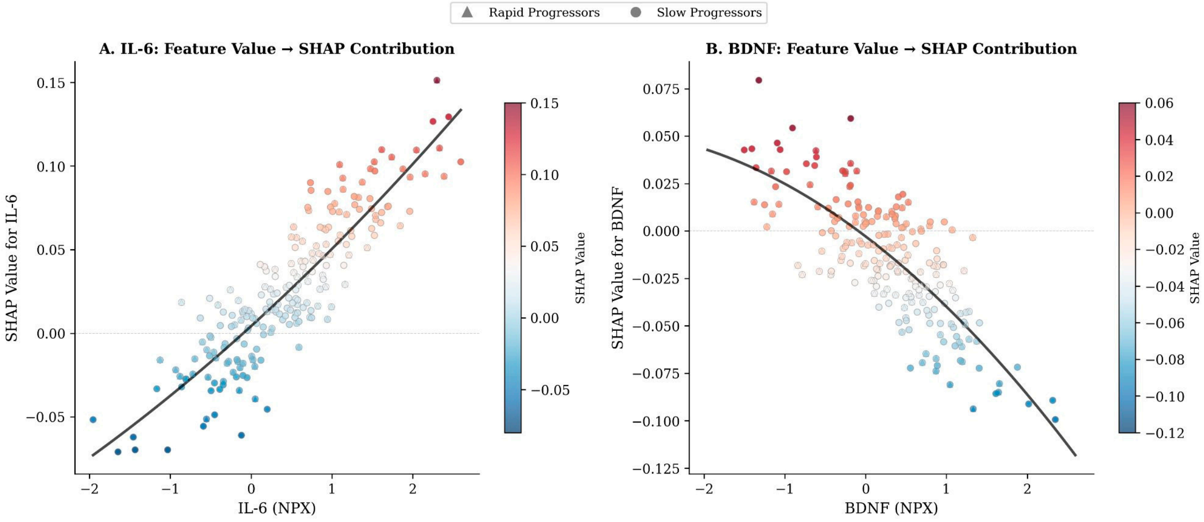

Figure S4. Feature value–SHAP contribution scatter plots for IL-6 (A) and BDNF (B). Each point represents a patient; color indicates SHAP value magnitude and direction. Triangle markers denote rapid progressors; circles denote slow progressors. (A) IL-6 shows a monotonically increasing relationship. (B) BDNF shows a non-linear inverse relationship. Black curves represent LOESS fits.

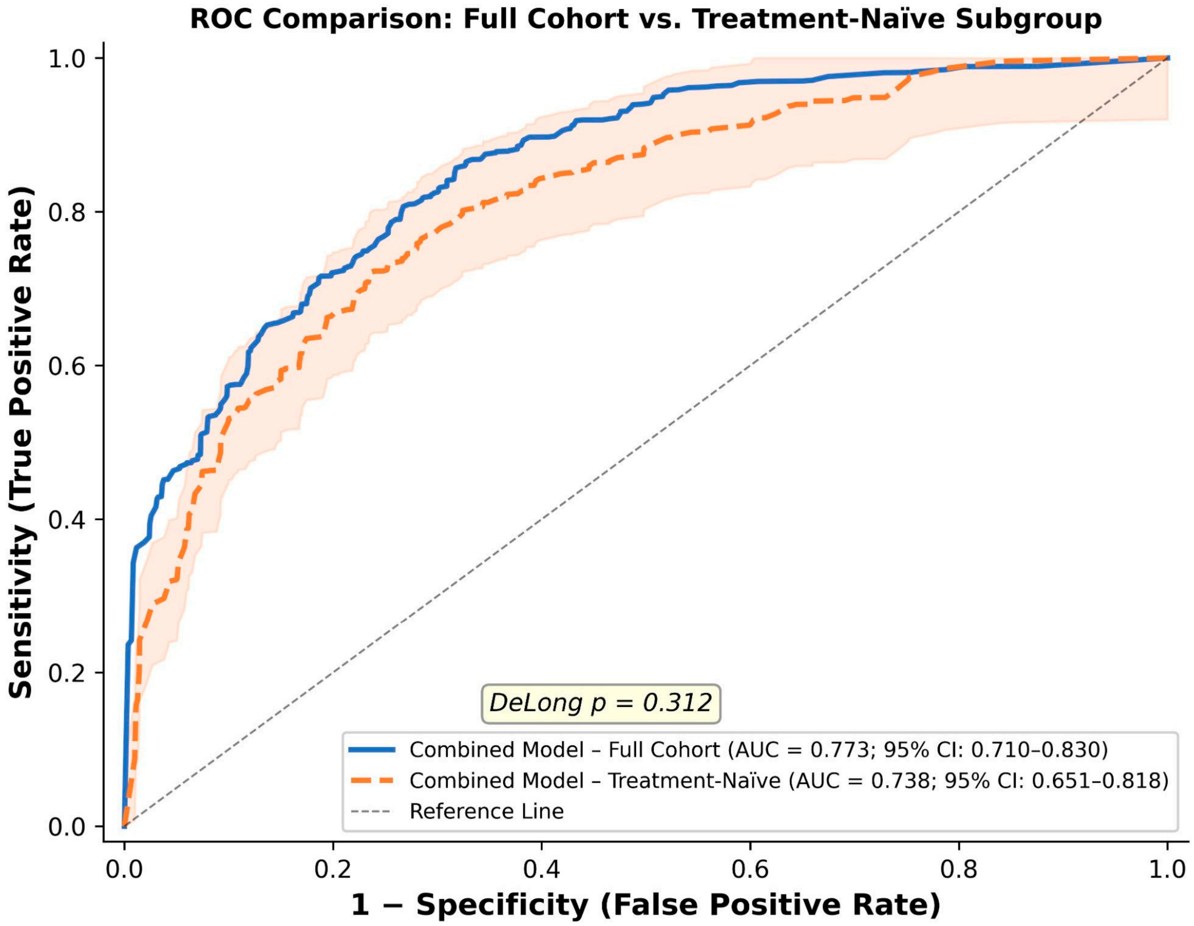

Figure S5. ROC comparison: full cohort vs. treatment-naïve subgroup. Combined model AUC 0.773 (95% CI: 0.710–0.830) in the full cohort and AUC 0.738 (95% CI: 0.651–0.818) in the treatment-naïve subgroup (n = 127). DeLong p = 0.312. Shaded regions represent 95% confidence bands.

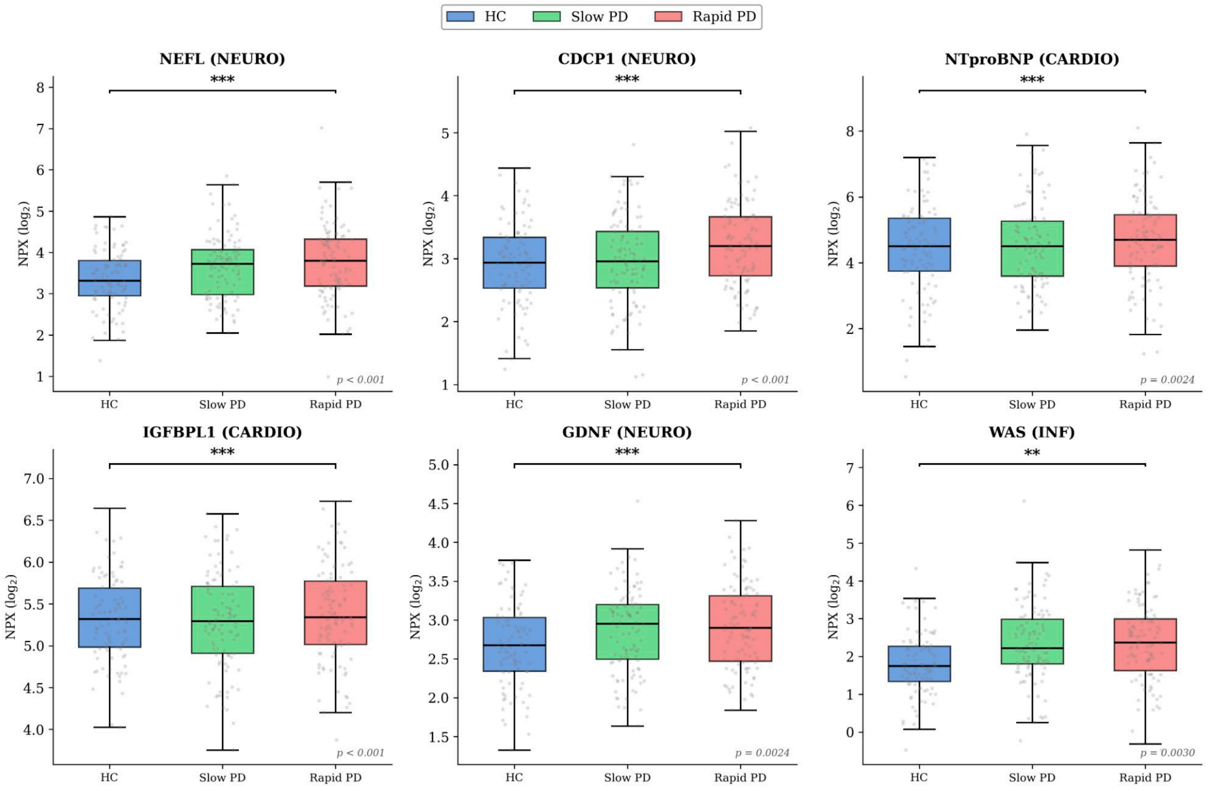

Figure S6. Three-group gradient analysis comparing plasma protein levels across healthy controls (HC, blue), slow progressors (green), and rapid progressors (red). NEFL, CDCP1, NTproBNP, IGFBPL1, and GDNF demonstrated significant monotonic gradients (HC → slow PD → rapid PD; all ANOVA $p < 0.001$). NPX, Normalized Protein eXpression (log_2 scale).
